# Pseudocapacitance of MXene nanosheets for high-power sodium-ion hybrid capacitors

**DOI:** 10.1038/ncomms7544

**Published:** 2015-04-02

**Authors:** Xianfen Wang, Satoshi Kajiyama, Hiroki Iinuma, Eiji Hosono, Shinji Oro, Isamu Moriguchi, Masashi Okubo, Atsuo Yamada

**Affiliations:** 1 Department of Chemical System Engineering, School of Engineering, The University of Tokyo, Hongo 7-3-1, Bunkyo-ku, Tokyo 113-8656, Japan; 2 National Institute of Advanced Industrial Science and Technology, Umezono 1-1-1, Tsukuba 305-8568, Japan; 3 Graduate School of Engineering, Nagasaki University, Bunkyo-machi 1-14, Nagasaki 852-8521, Japan; 4 Elements Strategy Initiative for Catalysts and Batteries (ESICB), Kyoto University, Nishikyo-ku, Kyoto 615-8510, Japan

## Abstract

High-power Na-ion batteries have tremendous potential in various large-scale applications. However, conventional charge storage through ion intercalation or double-layer formation cannot satisfy the requirements of such applications owing to the slow kinetics of ion intercalation and the small capacitance of the double layer. The present work demonstrates that the pseudocapacitance of the nanosheet compound MXene Ti_2_C achieves a higher specific capacity relative to double-layer capacitor electrodes and a higher rate capability relative to ion intercalation electrodes. By utilizing the pseudocapacitance as a negative electrode, the prototype Na-ion full cell consisting of an alluaudite Na_2_Fe_2_(SO_4_)_3_ positive electrode and an MXene Ti_2_C negative electrode operates at a relatively high voltage of 2.4 V and delivers 90 and 40 mAh g^−1^ at 1.0 and 5.0 A g^−1^ (based on the weight of the negative electrode), respectively, which are not attainable by conventional electrochemical energy storage systems.

## Abstract (web summary)

Conventional sodium ion batteries have low energy and power densities. Here, the authors report the use of MXene Ti_2_C as a negative electrode for sodium ion energy storage, and show that the pseudocapacitance of the electrode allows the hybrid capacitors to achieve high electrochemical performance.

## Graphical abstract

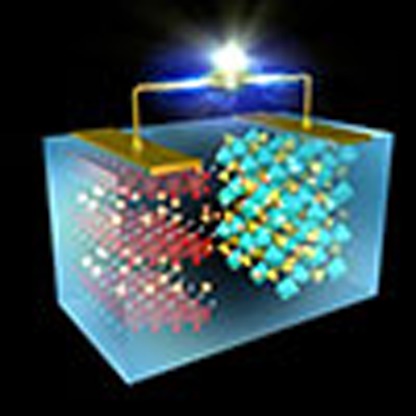

Electrochemical energy storage capable of more energy at high charge−discharge rates is receiving considerable attention because of the strong industrial demands for their widespread use in the smart grid. High-energy Li-ion batteries consisting of Li^+^ (de)intercalation electrodes currently power most portable electronics, but the power density is not satisfactory for high-power applications^1^,^2^. High-power electrochemical capacitors make up many markets ranging from electronics to transportation and stationary applications, but the low energy density is a serious disadvantage^3^,^4^. This trade-off between the power and energy densities of electrochemical energy storage has been well recognized, which intrinsically originates from the charge storage mechanism of the electrodes: ion (de)intercalation in batteries and double-layer formation in electrochemical capacitors. In general, ion (de)intercalation stores charge more slowly than a double layer, whereas a double layer stores less charge than ion (de)intercalation^3^. Thus, the pseudocapacitance (or redox capacitance) has become an increasingly important mechanism for charge storage^5^,^6^,^7^,^8^,^9^,^10^. Pseudocapacitance occurs through (i) monolayer ion adsorption on the electrode surface with charge transfer, (ii) redox at the electrode surface (RuO_2_·*x*H_2_O, ref. 5) or (iii) ultrafast ion intercalation into the bulk (*T*-Nb_2_O_5_, ref. 6), whereby charge storage is partially liberated from the trade-off.

At present, urgent concerns over lithium resources have focused recent attention on Na-ion batteries owing to the abundance and low cost of sodium. The present set of negative-electrode materials for Na-ion batteries mainly consists of Na-ion intercalation, alloying or conversion materials such as hard carbon, expanded graphite, TiO_2_, Na_2_Ti_3_O_7_, phosphorus, Sb_2_S_3_, Sn_4+*x*_P_3_, SnS_2_ and P2-Na_0.66_[Li_0.22_Ti_0.78_]O_2_ (refs 11, 12, 13, 14, 15, 16, 17, 18, 19). However, their energy and power densities severely suffer from the trade-off, and the large volume change accompanied by the reaction with the large Na ion generally results in poor cycle stability, both of which have spurred us to develop new negative electrodes based on pseudocapacitive charge storage for advanced Na-ion systems.

We have targeted MXenes as pseudocapacitor electrodes in this study. MXenes have been developed as a novel family of nanosheet compounds by Gogotsi, Barsoum and colleagues^20^,^21^,^22^,^23^,^24^,^25^ MXenes are chemically derived from layered *M*_*n*+1_*AX*_*n*_ or MAX phases, where *M* is an early transition metal, *A* is an A-group element and *X* is C and/or N^26^. For example, a hydrofluoric-acid treatment of Ti_3_AlC_2_ selectively extracts the aluminum layer, resulting in an exfoliated nanosheet compound, the MXene Ti_3_C_2_ (Ti_3_C_2_T_*x*_ where T_*x*_ denotes surface function groups), which has a surface terminated with OH, F and O. Importantly, the MXene has both a high electrical conductivity and large surface area; therefore, its application to pseudocapacitor electrodes holds great promise. Indeed, the pseudocapacitance of Ti_3_C_2_T_*x*_, Ti_2_CT_*x*_, V_2_CT_*x*_ and Nb_2_CT_*x*_ in nonaqueous Li-ion electrolytes delivers a specific capacity >100 mAh g^−1^ below 2 V versus Li/Li^+^ (refs 22, 27, 28, 29, 30, 31, 32).

Here, we report on Ti_2_CT_*x*_ as a negative electrode material for Na-ion hybrid capacitors. The pseudocapacitance of Ti_2_CT_*x*_ allows Na-ion hybrid capacitors to be liberated from the trade-off between high energy and high power.

## Results

### Electrochemical properties of Ti_2_CT_*x*_

Ti_2_CT_*x*_ was synthesized by treating the MAX phase Ti_2_AlC with a 10% HF aqueous solution. Powder X-ray diffraction (XRD) shows the expansion of the interlayer distance by the hydrofluoric acid (HF) treatment, suggesting successful removal of the Al layers by the HF treatment (Supplementary Fig. 1). The significant decrease in the amount of Al according to energy dispersive X-ray spectroscopy (EDX) supports the selective extraction of Al. A delamination process such as sonication was not conducted^22^; therefore, Ti_2_CT_*x*_ in this work is a multilayered MXene, as imaged by scanning electron microscopy (Supplementary Fig. 2).

**Figure 1:**
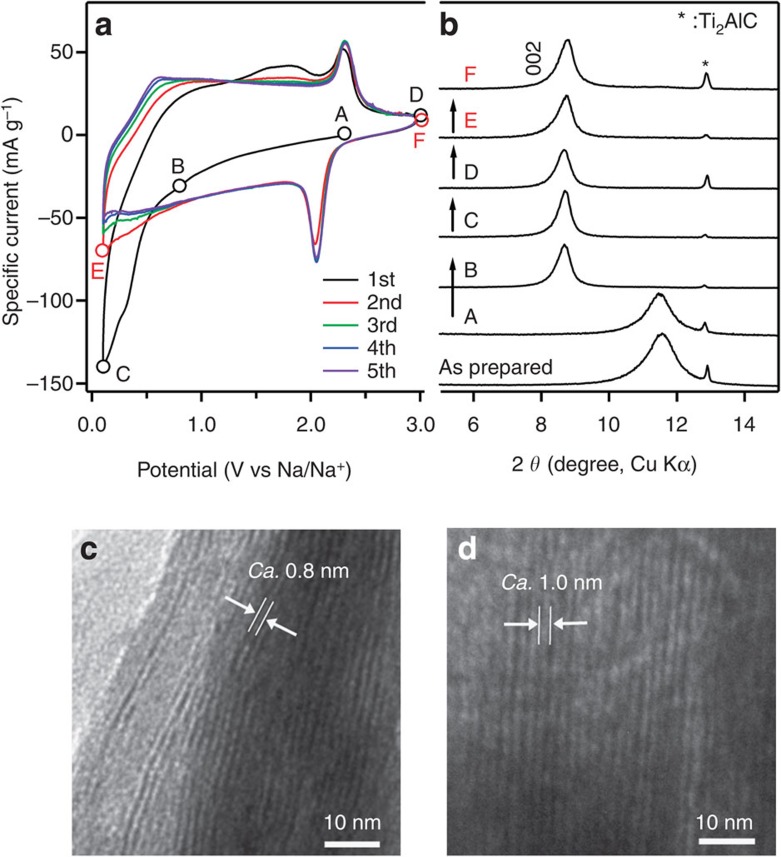
Electrochemical properties and associated structural changes of the Ti_2_CT_*x*_. (**a**) CV for Ti_2_CT_*x*_ in a 1 M NaPF_6_/EC−DEC electrolyte at a scan rate of 0.2 mV s^−1^. (**b**) *Ex situ* XRD patterns during the CV cycles. (**c**) Transmission electron microscopy (TEM) image of the pristine Ti_2_CT_*x*_. (**d**) TEM image of activated Ti_2_CT_*x*_ after the first CV.

**Figure 2:**
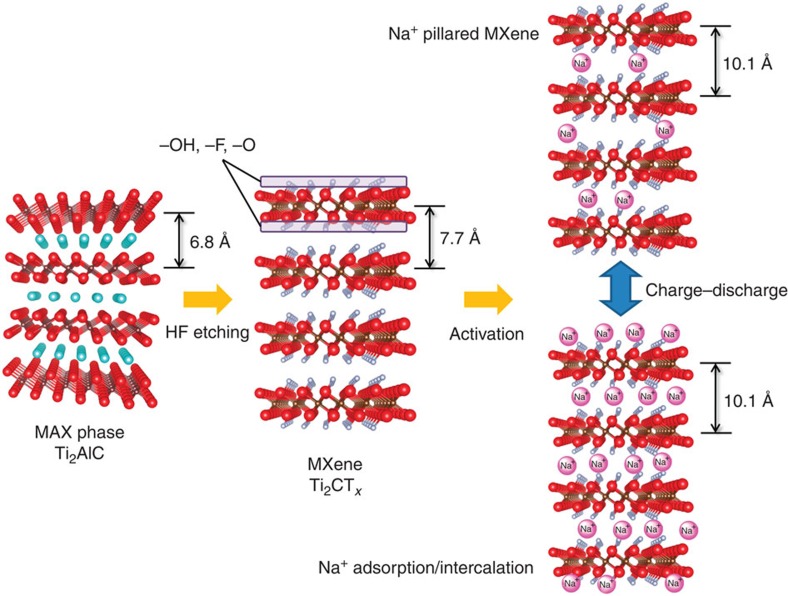
Schematic illustration of the reaction mechanism of Ti_2_CT_*x*_ by electrochemical activation. The MAX phase Ti_2_AlC is transformed to the MXene Ti_2_CT_*x*_ nanosheets by the hydrofluoric acid treatment. Ti_2_CT_*x*_ exhibits expansion of the interlayer distance by the first Na^+^ intercalation, then reversible Na^+^ (de)intercalation occurs without significant interlayer distance change.

The Ti_2_CT_*x*_ electrode in a nonaqueous Na^+^ electrolyte behaves differently than in an aqueous Na^+^ electrolyte or a nonaqueous Li^+^ electrolyte. Figure 1a shows the cyclic voltammetry (CV) and corresponding *ex situ* XRD patterns of the MXene Ti_2_C in a 1 M NaPF_6_/ethylene carbonate (EC)−diethyl carbonate (DEC) electrolyte. Lukatskaya *et al.*^23^ reported that Ti_3_C_2_T_*x*_ placed in various aqueous salt (NaOH, KOH, LiOH and so on) solutions exhibits a downshift in the (002) diffraction peak in the XRD pattern, corresponding to spontaneous cation intercalation. In contrast, Ti_2_CT_*x*_ immersed in a nonaqueous 1 M NaPF_6_/EC−DEC electrolyte exhibits no downshift in the (002) diffraction peak by immersion into the electrolyte alone, which indicates that Na^+^ intercalation does not occur spontaneously. The first cathodic scan exhibits an irreversible current below 1.0 V versus Na/Na^+^ and an associated downshift in the (002) diffraction peak. As EDX confirms an increase in the Na content in Ti_2_CT_*x*_ (Supplementary Table S1), Na^+^ is definitely intercalated/adsorbed into/onto the MXene sheets electrochemically. On the basis of the shift in the (002) diffraction peak, the expansion of the interlayer distance is estimated as 2.5 Å. The expansion is also confirmed by transmission electron microscopy images (Fig. 1b,c). Because the expansion is consistent with the size of bare Na^+^, Na^+^ intercalation occurs after desolvation at the electrode/electrolyte interface during the initial reduction process (Fig. 2, activation).

After initial Na^+^ intercalation, Ti_2_CT_*x*_ exhibits pseudocapacitor behaviour. The subsequent CV cycles after the first cycle exhibit the stable rectangular-shaped CV below ~2.0 V, corresponding to typical capacitor behaviour. In addition, a reversible pair of relatively sharp cathodic/anodic peaks is observed at ~2.3 V in each cycle. Although the (002) diffraction peak does not shift during the cycles, EDX confirms a reversible change in the Na content, and X-ray photoelectron spectroscopy (XPS) detects a reversible spectral change in the Ti 2*p* XPS spectrum (Supplementary Fig. 3), which is indicative of a charge transfer reaction. Therefore, the current flow from Ti_2_CT_*x*_ in the nonaqueous Na^+^ electrolyte is attributable to the pseudocapacitance without exhibiting shrinkage/expansion of the interlayer distance.

As reported previously, Ti_3_C_2_T_*x*_ and Ti_2_CT_*x*_ in a nonaqueous Li^+^ electrolyte exhibit shrinkage/expansion of the interlayer distance during the cycle^27^,^28^. For example, Ti_2_CT_*x*_ in a nonaqueous Li^+^ electrolyte exhibits a change in the interlayer distance between 18.7 and 18.0 Å (4% change). In contrast, bare Na^+^ is intercalated between the MXene sheets for Ti_2_CT_*x*_ in a nonaqueous Na^+^ electrolyte to expand the interlayer distance from 7.7 to 10.1 Å during the first cathodic process (Fig. 2, activation). Then, activated Ti_2_CT_*x*_ allows reversible Na^+^ intercalation/deintercalation into the interlayer space as well as reversible Na^+^ adsorption/desorption onto the surface of each layer/sheet, where the change in the interlayer distance is small within ~0.1 Å (Fig. 2 and Supplementary Table 2). Because EDX detects residual Na^+^, even at 3.0 V (Supplementary Table 1), residual Na^+^ may behave as a pillar between the MXene Ti_2_C layers during the entire charge/discharge process, maintaining the interlayer distance at an almost constant value. Another possible explanation for the constant interlayer distance is that the expansion due to Na^+^ intercalation is balanced by the Coulombic attraction between the MXene sheets and intercalated Na^33^.

The CV curves of activated Ti_2_CT_*x*_ measured at various scan rates (Supplementary Fig. 4) provide insights into the reaction kinetics. The *b*-value analysis (*i*=*av*^*b*^; *i*: current; *v*: scan rate; *a*, *b*: constants) for the peak current at ~2.3 V gives a *b*-value of unity for 0.05<*v*<0.5 mV s^−1^, which is indicative of a surface-controlled reaction. Presumably, (de)solvation or charge transfer at the interface rather than slow Na^+^ diffusion in the bulk determines the reaction rate.

### Ti_2_CT*_x_* as negative-electrode material

**Figure 3:**
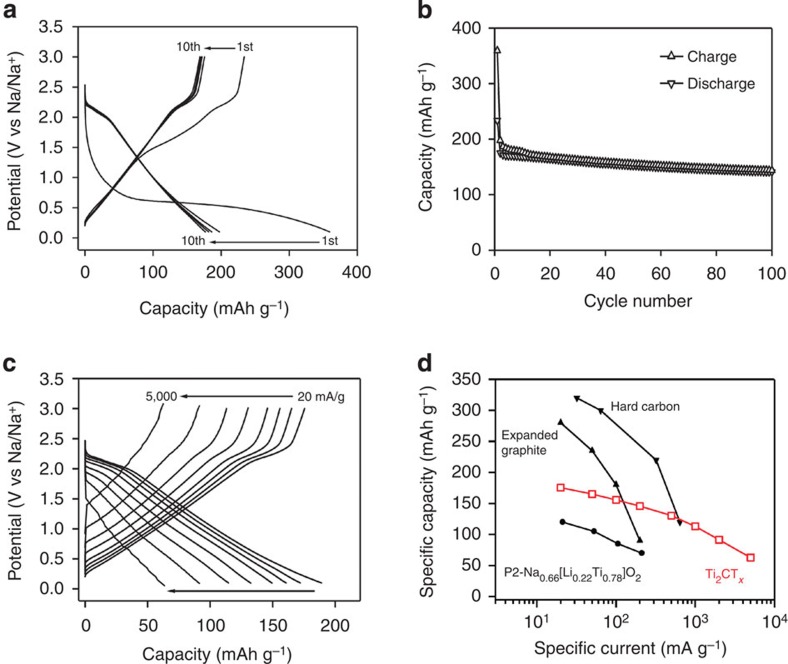
Electrode performance of Ti_2_CT_*x*_ in a Na^+^ nonaqueous electrolyte. (**a**,**b**) Charge/discharge curves and cycle stability for Ti_2_CT_*x*_ in a 1 M NaPF_6_/EC−DEC electrolyte. The specific current is 20 mA g^−1^ with cut-off voltages in the range of 0.1−3.0 V versus (vs) Na/Na^+^. (**c**) Charge/discharge curves at various rates. (**d**) Rate capability for Ti_2_CT_*x*_, hard carbon^11^, expanded graphite^12^ and P2-Na_0.66_[Li_0.22_Ti_0.78_]O_2_ (ref. 19).

Having confirmed that activated Ti_2_CT_*x*_ is a promising candidate as a negative-electrode material for Na-ion hybrid capacitors, galvanostatic charge/discharge experiments were conducted. Here, charging is a cathodic process (Na^+^ adsorption/intercalation), whereas discharging is an anodic process (Na^+^ desorption/deintercalation). Figure 3a shows the charge−discharge curves measured between the cut-off voltages of 0.1−3.0 V versus Na/Na^+^ at 20 mA g^−1^. The first charge exhibits a voltage plateau at ~0.6 V, delivering a relatively large capacity of 360 mAh g^−1^. The following first discharge curve has a capacitor-type slope in the range of 0.2−2.5 V but with a much smaller capacity, resulting in a relatively low Coulombic efficiency of 65% for the first cycle. However, after the initial few cycles for activation, Ti_2_CT_*x*_ exhibits stable and efficient electrode performance. The charge/discharge profiles exhibit a capacitor-type slope in the range of 0.1−2.3 V (an average operating potential of 1.3 V), delivering a reversible capacity of ~175 mAh g^−1^ with good cycle stability (Fig. 3b, 12 and 19% losses of the second capacity after 50 and 100 cycles, respectively). The average operating potential of 1.3 V is relatively high when used as the negative electrode but is beneficial for stable operation, retaining the capacity at a high rate and avoiding Na metal plating for safety.

The Na/Ti elemental ratio determined by EDX during the first two cycles allow us to estimate the capacity (Supplementary Fig. 5) for various possible nominal chemical formulae of Ti_2_CT_*x*_ (Ti_2_CO_2_, Ti_2_C(OH)_2_ and Ti_2_CF_2_). The first experimental charge capacity (359 mAh g^−1^) is larger than the estimated ones (308−321 mAh g^−1^), whereas the first discharge capacity (233 mAh g^−1^) is close to the estimated ones (224−234 mAh g^−1^). Thus, the initial irreversible capacity arises from the decomposition of the electrolyte as well as the residual Na^+^ between the MXene layers (*vide supra*). The electrolyte decomposition during the initial cycles may form stable solid−electrolyte interface, which inhibits continuous electrolyte decomposition.

Figure 3c shows the charge/discharge profiles of Ti_2_CT_*x*_ at various specific currents. The reversible capacity is retained, even at extremely high rates (156, 113 and 63 mAh g^−1^ at 200, 1,000 and 5,000 mA g^−1^, respectively). Figure 3d compares the rate capability of Ti_2_CT_*x*_, hard carbon^11^, expanded graphite^12^ and P2-Na_0.66_[Li_0.22_Ti_0.78_]O_2_ (ref. 19). Although the carbon compounds (expanded graphite and hard carbon) deliver a high capacity of ~300 mAh g^−1^ at a low rate, the capacity retention at high rates is much lower than that of Ti_2_CT_*x*_. P2-Na_0.66_[Li_0.22_Ti_0.78_]O_2_ is an electrode material without a change in lattice volume during the cycle; therefore, the minimum structural change associated with (de)sodiation enables a high capacity retention at high rates. However, the theoretical capacity of P2-Na_0.66_[Li_0.22_Ti_0.78_]O_2_ is much smaller than that of Ti_2_CT_*x*_; Ti_2_CT_*x*_ delivers much higher capacity at any charge/discharge rate. Therefore, Ti_2_CT_*x*_ is a high-performance electrode material with a high capacity, stability, safety and a high power. It should be emphasized that the high rate capability was obtained with a thick electrode (thickness: 50 μm); therefore, its practical application to Na-ion hybrid capacitors is highly realistic.

### Full cell with Ti_2_CT_x_ and Na_2_Fe_2_(SO_4_)_3_

**Figure 4:**
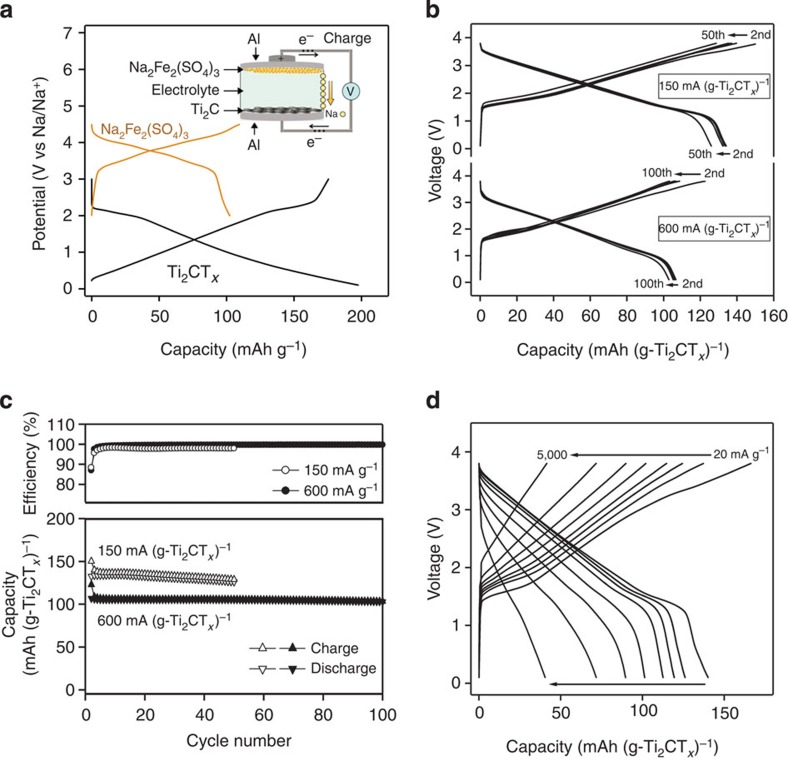
Performance of the prototype Na-ion hybrid capacitor full cells. (**a**) Charge/discharge curves of Ti_2_CT_*x*_ and alluaudite Na_2_Fe_2_(SO_4_)_3_ versus Na/Na^+^; the specific currents are 30 and 6 mA g^−1^, respectively. The inset is a schematic illustration of the Ti_2_CT_*x*_−alluaudite Na_2_Fe_2_(SO_4_)_3_ full cell. (**b**) Voltage profile of the Ti_2_CT_*x*_−alluaudite Na_2_Fe_2_(SO_4_)_3_ full cell at the specific currents of 150 and 600 mA (g−Ti_2_CT_*x*_)^−1^. The specific capacity is normalized by the weight of Ti_2_CT_*x*_. (**c**) Cycle stability and Coulombic efficiency of the full cell with cutoff voltages in the range of 0.1−3.8 V at rates of 150 and 600 mA (g−Ti_2_CT_*x*_)^−1^. (**d**) Charge/discharge profiles at various rates.

To further demonstrate the potential of Ti_2_CT_*x*_ in Na-ion hybrid capacitors, a prototype full cell (Fig. 4a) was fabricated by utilizing Ti_2_CT_*x*_ as a negative electrode and alluaudite Na_2_Fe_2_(SO_4_)_3_ as a positive electrode. Recently, our group has discovered alluaudite Na_2_Fe_2_(SO_4_)_3_ as a promising positive-electrode material that exhibits a specific capacity of 100 mAh g^−1^ at a high operating potential of 3.8 V versus Na/Na^+^, the highest value among all Fe-based compounds, which enables the Na-ion full cell to achieve both high energy and high power densities^34^,^35^. The mass balance between the positive and negative electrodes was fixed to 4:1 to ensure full activation of Ti_2_CT_*x*_ during the first charge.

Figure 4b shows the charge/discharge curves for the full cell measured at current densities of 150 and 600 mA g^−1^ (based on the weight of Ti_2_CT_*x*_). Note that the first cycle was conducted at 20 mA g^−1^ (based on the weight of Ti_2_CT_*x*_) to activate Ti_2_CT_*x*_. After the first cycle, the cell exhibits a voltage profile that mainly reflects the potential of Ti_2_CT_*x*_ because the cell capacity is limited by the Ti_2_CT_*x*_ negative electrode (Fig. 3a). The average operating voltage is 2.4 V, and the reversible capacities of the second cycle are 133 and 107 mAh g^−1^ at 150 and 600 mA g^−1^ (based on the weight of Ti_2_CT_*x*_), respectively, achieving theoretical energy densities of 320 and 260 Wh kg^−1^ at specific power densities of 360 and 1,440 W kg^−1^ (based on the weight of Ti_2_CT_*x*_), respectively.

Both the cycle stability and efficiency of the full cell are extremely high. As demonstrated in Fig. 4c, the loss of the discharge capacity after 100 cycles at 600 mA g^−1^ is only 4% of the second discharge capacity. Furthermore, the Coulombic efficiency for cycling at 600 mA g^−1^ reaches ~99.7% after a few initial cycles. The excellent cycle stability and efficiency may be attributed to the pseudocapacitive charge storage mechanism without a large structural change.

Figure 4d plots the charge/discharge profiles of the alluaudite Na_2_Fe_2_(SO_4_)_3_−Ti_2_CT_*x*_ full cell at various current rates, demonstrating the promising high-power performance of the cell (Supplementary Fig. 6). The full cell provides reversible capacities of 90 and 40 mAh g^−1^, even at extremely high rates of 1,000 and 5,000 mA g^−1^ (based on the weight of Ti_2_CT_*x*_). Thus, by utilizing the pseudocapacitive negative electrode, the Na-ion hybrid capacitor overcomes the trade-off limit and potentially surpasses state-of-the-art high-power rechargeable batteries. Although the capacity for the total weight of the positive and negative electrode materials in the present prototype cell is limited (26.6 mAh g^−1^ at a low rate) owing to the excess amount of positive-electrode material, an improvement in the initial Coulombic efficiency could reduce the amount of positive-electrode material, and hence, greatly increase the capacity of the full cell.

## Discussion

Finally, we note a positive perspective for further improvement in the Ti_2_CT_*x*_ electrode. The gravimetric capacitance from CV of the Ti_2_CT_*x*_ electrode in this work is higher than that of previously reported Ti_3_C_2_T_*x*_ electrodes because of the light weight of Ti_2_CT_*x*_ (Supplementary Fig. 7). However, very recently, Ghidui *et al.*^36^ have reported a new method to synthesize Ti_3_C_2_T_*x*_ (Ti_3_C_2_T_*x*_ ‘clay’) using a solution of lithium fluoride and hydrochloric acid. The resulting Ti_3_C_2_T_*x*_ ‘clay’ delivers a high volumetric capacitance of 900 F cm^−3^ and a gravimetric capacitance of 245 F g^−1^, which is much higher than those of the previously reported Ti_3_C_2_T_*x*_ electrodes and the Ti_2_CT_*x*_ electrode in this work. According to Ghidui *et al.*^36^, the Ti_3_C_2_T_*x*_ clay provides a much higher capacitance at higher rates owing to the improved accessibility of the interlayer spacing. Therefore, the synthetic procedure applied for the Ti_3_C_2_T_*x*_ clay, that is, the LiF+HCl treatment, could further improve the performance of Ti_2_CT_*x*_.

In summary, the use of the pseudocapacitance of MXene nanosheets was demonstrated as an effective strategy for developing high-performance Na-ion hybrid capacitors. A pseudocapacitance with no significant structural changes can store more charge at a faster charge–discharge rate relative to the ion intercalation and double-layer mechanisms. In the present case, Ti_2_CT_*x*_ operates as a pseudocapacitor electrode material at 1.3 V on average versus Na/Na^+^ with a reversible capacity of 175 mAh g^−1^. The alluaudite Na_2_Fe_2_(SO_4_)_3_−Ti_2_CT_*x*_ prototype cell delivers a high specific energy of 260 Wh kg^−1^ at a high specific power of 1.4 kW kg^−1^ (based on the weight of Ti_2_CT_*x*_), which overcomes the trade-off limit associated with conventional electrochemical energy storage. By exploiting the compositional and structural versatility of MAX phases and the resulting optimized MXenes, we could further extend the possible sets of electrodes for advanced Na-ion hybrid capacitors.

## Methods

### Synthesis of Ti_2_CT_*x*_

Ti_2_AlC was prepared by heating a precursor mixture of TiC (>99%, High Purity Chemicals, Japan), Ti (>99%, High Purity Chemicals, Japan) and Al (>99.9%, High Purity Chemicals, Japan) at 1,300 °C for 1 h in an Ar gas environment. Ti_2_CT_*x*_ was synthesized by treating 1 g of Ti_2_AlC powder in 10% HF aqueous solution (Wako) for 12 h at room temperature. The HF-treated powder was dried in vacuum at 60 °C for 24 h.

### Materials characterization

Powder X-ray diffraction patterns were recorded on a Rigaku RINT-TTR III powder diffractometer with Cu Kα radiation in a step of 0.02° over a 2*θ* range of 10−80°. Scanning electron microscopy measurements were carried out on a JEOL 6510FA apparatus equipped with an energy dispersive X-ray (EDX) spectrometer for chemical analysis. Transmission electron microscopy was conducted on a JEOL JEM-2100 at 200 kV. X-ray photoelectron spectroscopy (XPS) data were collected using a ULVAC PHI 5000 VersaProbe spectrometer with monochromatized Al Kα radiation (*hv*=1,486.6 eV). The pressure in the analysis chamber during the measurements was maintained in the 10^−7^ Pa range. The powder sample was pressed onto conductive carbon, and peaks were recorded with a constant pass energy mode of 117 eV for survey investigation. High-resolution spectra were taken at a pass energy of 23 eV, with a step of 0.1 eV. All binding energies were referenced to that of free carbon at 284.5 eV. The crystal structure was drawn using VESTA^37^.

### Electrochemical measurement

For electrochemical studies, the working electrode was fabricated by mixing 80 wt% Ti_2_CT_*x*_, 10 wt% acetylene black and 10 wt% polyvinylidene difluoride binder in a minimal amount of *N*-methylpyrrolidone (NMP) solvent. This slurry was pasted on an aluminum-foil current collector, and the as-obtained electrode sheet was dried overnight at 120 °C in vacuum. The electrode thickness and mass loading are ~50 μm and ~1 mg cm^−2^, respectively, for all electrochemical measurements (CV and charge–discharge). CR2032-type coin cells were assembled with Na metal as the counter electrode, a glass fibre filter (GB-100R, ADVANTEC) as the separator and 1 M NaPF_6_ in ethylene carbonate-diethyl carbonate (EC−DEC, 1:1 v/v%) as the electrolyte. These cells were assembled inside an Ar-filled glove box (Miwa Inc., Japan) (dew point <−100 K). They were galvanostatically (dis)charged in the potential range 0.1–3.0 V. The gravimetric capacitance in farads per gram from CV is given by 
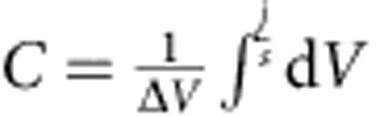
, where *C* is the gravimetric capacitance, *j* is the specific current in amperes per gram, *s* is the scan rate in volts per second, *V* is the voltage in volts and Δ*V* is the voltage window.

### Full cell fabrication

For full cell preparation, alluaudite Na_2_Fe_2_(SO_4_)_3_ was used as a positive electrode material. Alluaudite Na_2_Fe_2_(SO_4_)_3_ was synthesized according to the procedure described previously^34^. The full cell was prepared with the alluaudite Na_2_Fe_2_(SO_4_)_3_ electrode as a positive electrode, the Ti_2_CT_*x*_ electrode as a negative electrode, a glass fibrr membrane as a separator, and 1 M NaPF_6_/EC−DEC as an electrolyte. The cut-off voltages were 3.8 V for charging and 0.1 V for discharging.

## Author contributions

M.O. and A.Y. conceived and directed the project. M.O., X.W., S.K., H.I., E.H., S.O. and I.M. synthesized Ti_2_CT_*x*_. M.O. and X.W. measured and analysed the electrochemical properties. S.K. measured the performance of the full cells. All authors contributed to the writing of the paper.

## Additional information

**How to cite this article:** Wang, X. *et al.* Pseudocapacitance of MXene nanosheets for high-power sodium-ion hybrid capacitors. *Nat. Commun.* 6:6544 doi: 10.1038/ncomms7544 (2015).

## Supplementary Material

Supplementary Information Supplementary Figures 1-7, Supplementary Tables 1-2.
