# Supplementary material for: Pseudocapacitance of MXene nanosheets for high-power sodium-ion hybrid capacitors
Source: Nat Commun. 2015 Apr 2;6:6544. doi: 10.1038/ncomms7544 (PMC4396360; doi:10.1038/ncomms7544)
Supplement: Supplementary Information — Supplementary Figures 1-7, Supplementary Tables 1-2. [file ncomms7544-s1.pdf]

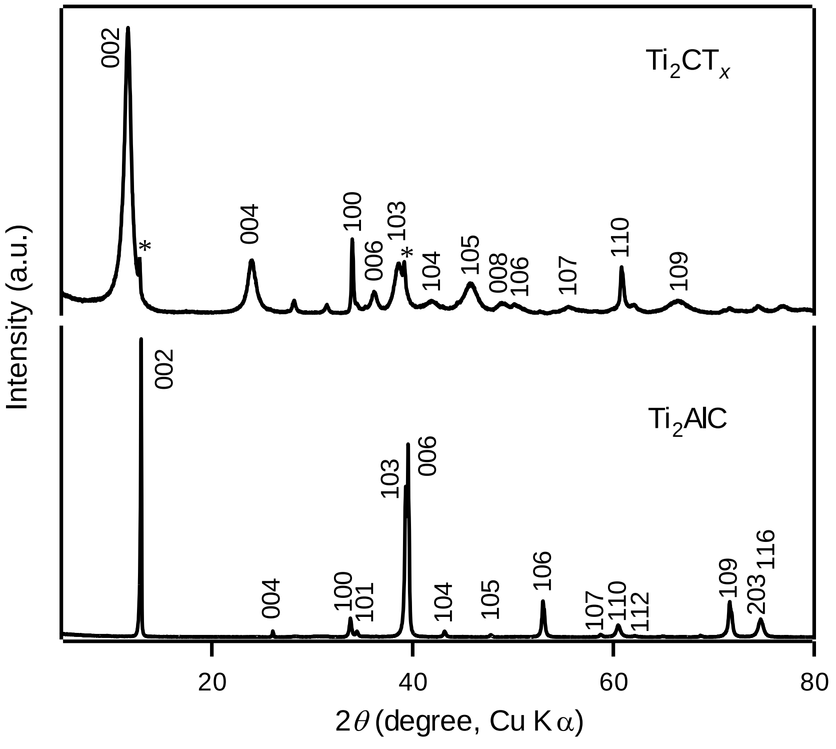

Supplementary Figure 1. Powder X-ray diffraction patterns for Ti_2AlC and Ti_2CT_x.

(a)

(b)

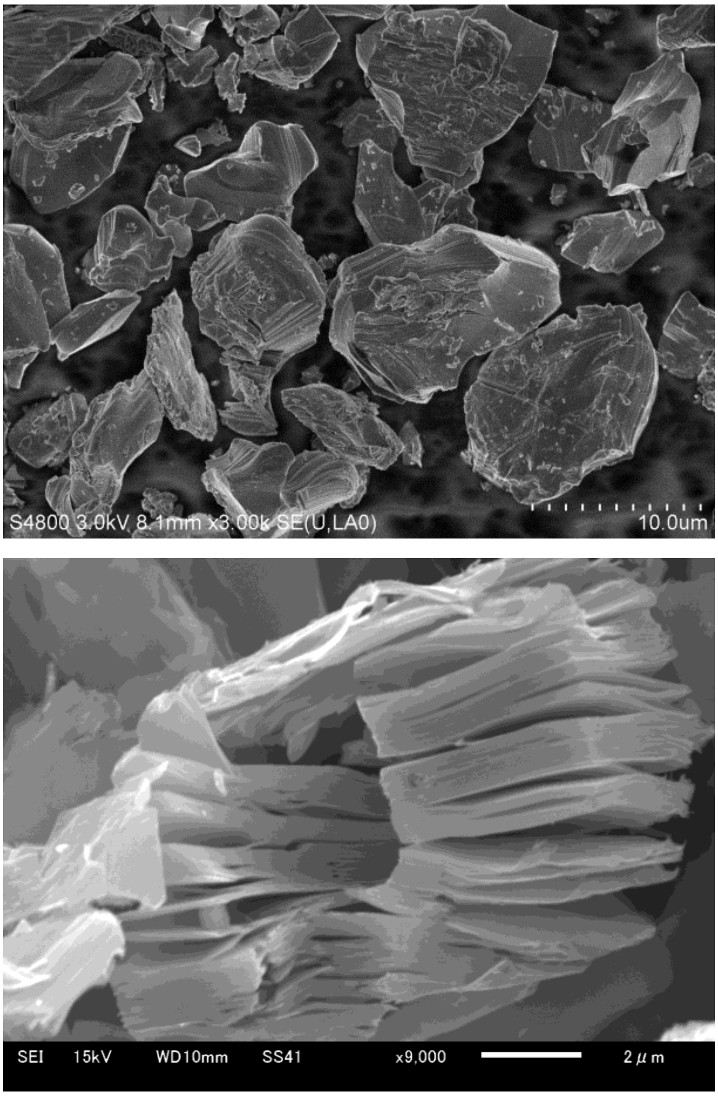

Supplementary Figure 2. SEM images for (a) Ti_2AlC and (b) Ti_2CT_x.

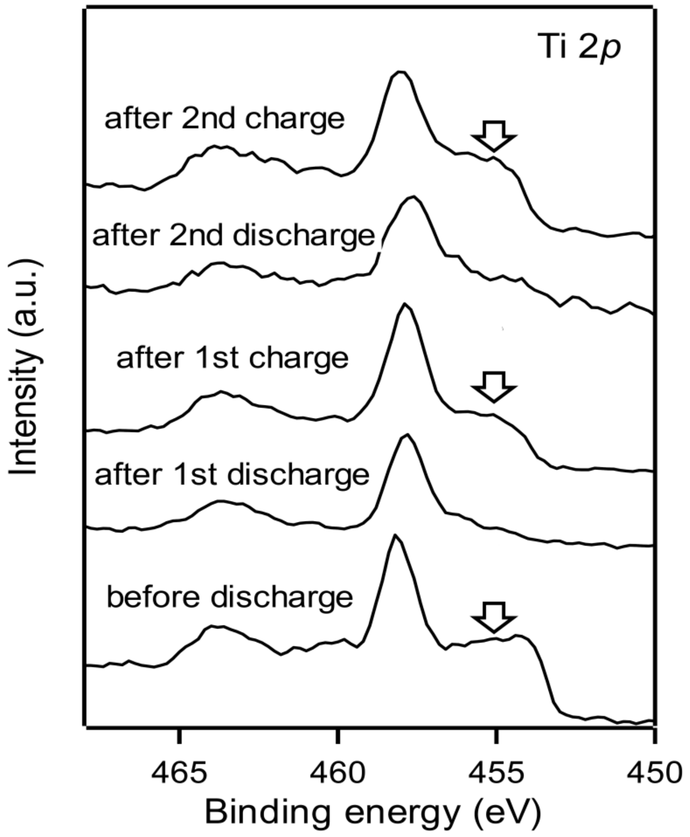

Supplementary Figure 3. *Ex situ* XPS spectra of Ti_2CT_x during charge−discharge.

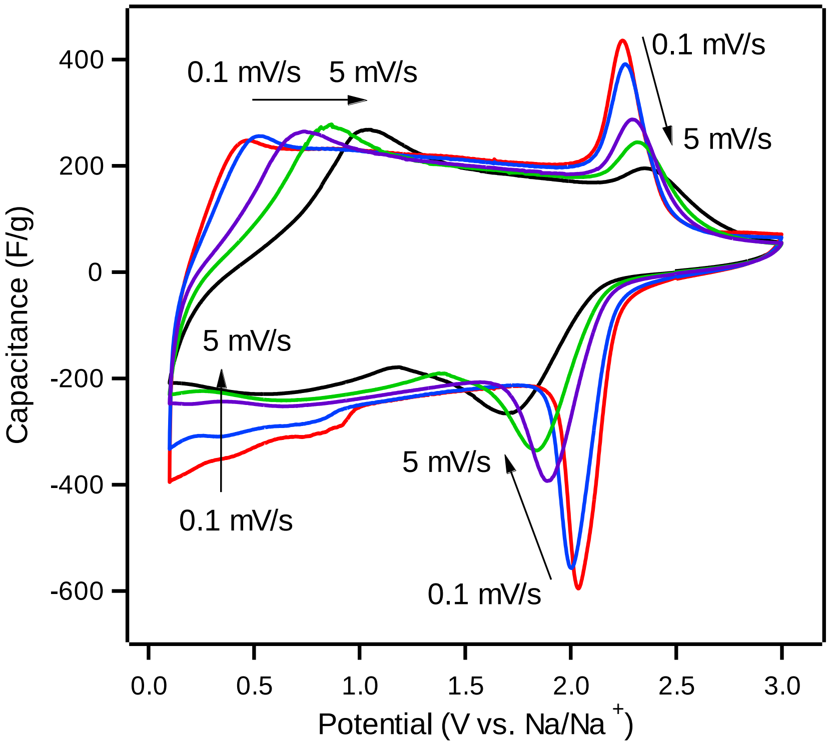

Supplementary Figure 4. Cyclic voltammetry curves at various scan rates. The thickness of the film electrode is *ca*. 50 μm. The gravimetric capacitance in F g^{-1} is calculated by *j*/*s* where *j* is the specific current [A g^{-1}] and *s* is the sweep rate [V s^{-1}].

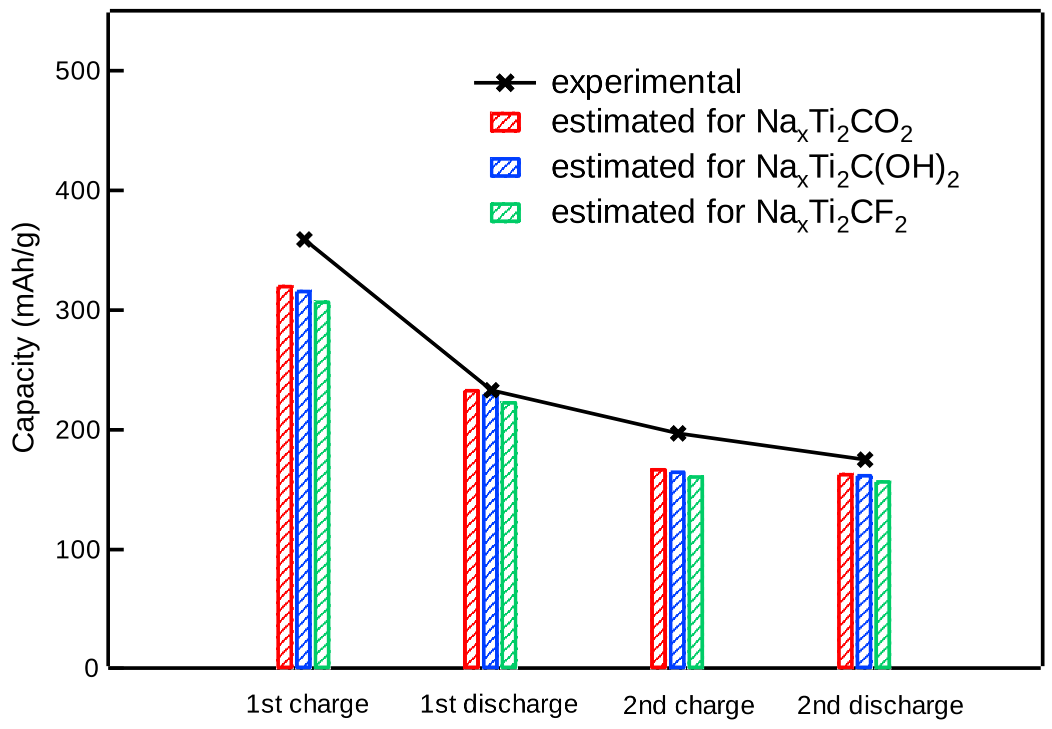

Supplementary Figure 5. Comparison between the experimental charge-discharge capacity and estimated capacity based on the Na/Ti ratio from EDX. The possible nominal chemical formulas of Ti_2CT_x (Ti_2CO_2, $Ti_2C(OH)_2$, and Ti_2CF_2) were assumed to estimate the capacity.

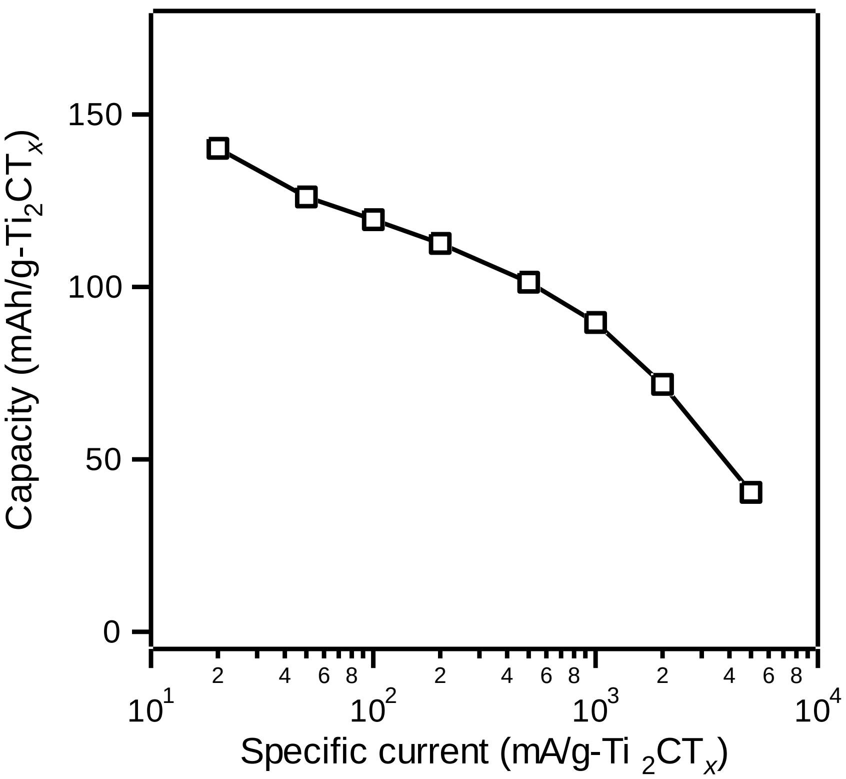

Supplementary Figure 6. Rate capability of Ti_2CT_x–*alluaudite* $Na_2Fe_2(SO_4)_3$ full cell.

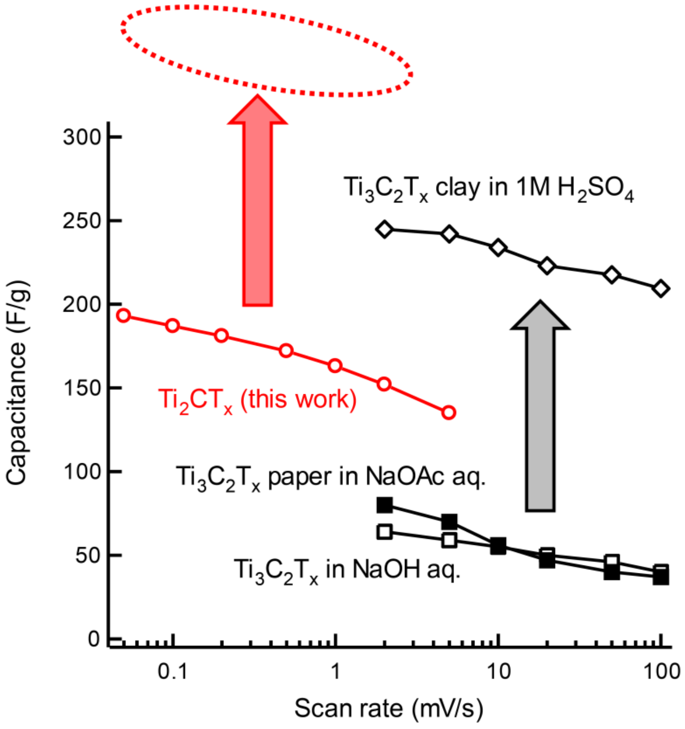

Supplementary Figure 7. Comparison of the rate performance of Ti_2CT_x in nonaqueous Na^+ electrolyte, and previously reported $Ti_3C_2T_x$ electrodes[23,36]. The gravimetric capacitance in F g^{-1} from the cyclic voltammetry is given by $C = \frac{1}{\Delta V}\int \frac{j}{s} dV$, where C is the gravimetric capacitance, j is the specific current in A g^{-1}, s is the scan rate [V s^{-1}], V is the voltage in V, and ΔV is the voltage window. The red arrow indicates a perspective of further improvement of the Ti_2CT_x electrode by adopting the new synthetic procedure in ref. 36.

Supplementary Table 1. Atomic ratio of Na and Ti in MXene Ti_2C electrode during the initial two cycles.

	Na/Ti
1st charge	0.84
1st discharge	0.23
2nd charge	0.67
2nd discharge	0.24

Supplementary Table 2. *c* parameters calculated from the (002) peak during charge and discharge.

MXene	electrolyte	state	Na^+ content	interlayer distance / Å	*c* / Å	reference
Ti_2CT_x	$NaPF_6$/EC-DEC	as prepaerd	0.0	7.65	15.30	This work
		electrolyte immersion	0.0	7.71	15.42	This work
		0.7 V at 1st charge	0.3	10.20	20.39	This work
		0.1 V at 1st charge	1.7	10.08	20.16	This work
		3.0 V at 1st discharge	0.5	10.21	20.42	This work
		0.1 V at 2nd charge	1.3	10.14	20.29	This work
		3.0 V at 2nd discharge	0.5	10.11	20.21	This work

MXene	electrolyte	state	Li^+ content	interlayer distance / Å	*c* / Å	reference
Ti_2CT_x	$LiPF_6$/EC-DMC	as prepared	0.0	7.73	15.46	Ref. [28]
		0.05 V at 1st charge	1.6	9.36	18.72	Ref. [28]
		2.7 V at 1st discharge	0.8	8.98	17.96	Ref. [28]
